# Noninvasive salivary biomarkers (PTX3, calprotectin, and IL-8) for early-onset neonatal pneumonia: case-control differences and exploratory discrimination

**DOI:** 10.3389/fped.2026.1747967

**Published:** 2026-03-04

**Authors:** Yan-Nan Wang, Lai-Shuan Wang

**Affiliations:** 1 Pediatric Department, The First People’ s Hospital of Jiashan, Jiaxing, Zhejiang, China; 2 Neonatology Department, Children’s Hospital of Fudan University, Shanghai, Jiangsu, China

**Keywords:** calprotectin, early-onset neonatal pneumonia, interleukin-8, pentraxin 3 (PTX3), salivary biomarkers

## Abstract

**Objective:**

Early-onset neonatal pneumonia (EONP) demands rapid recognition, but blood tests are invasive and may be delayed. This study evaluated whether noninvasive salivary pentraxin-3 (PTX3), calprotectin, and interleukin-8 (IL-8) differ between EONP and healthy controls and whether they reflect systemic inflammation.

**Methods:**

EONP required respiratory distress within 72 h of birth, new infiltrates on chest radiograph and/or lung ultrasound, and ≥1 laboratory or microbiologic criterion: abnormal leukocyte indices (I/T ratio > 0.16 or WBC/differential abnormality), hs-CRP ≥ 10 mg/L, PCT ≥ 0.5 ng/mL, or a positive blood/upper-airway culture with a compatible pathogen. Saliva was collected after definitive EONP diagnosis and immediately before systemic antibiotics in 100 EONP infants and 126 healthy controls. Biomarkers (PTX3, calprotectin, IL-8) were quantified by ELISA.

**Results:**

EONP infants had higher salivary PTX3 (median 2.11 *vs.* 0.79 ng/mL), calprotectin (11.65 *vs.* 3.07 ng/mL), and IL-8 (15.02 *vs.* 4.67 pg/mL) than healthy controls. After adjustment, calprotectin and IL-8 remained independently associated with EONP, whereas PTX3 did not retain statistical significance. In case—control discrimination, using ROC-derived cut-offs, AUCs were 0.865 (PTX3), 0.967 (calprotectin), and 0.930 (IL-8); a combined three-marker model achieved AUC 0.978. Within EONP, salivary PTX3, calprotectin, and IL-8 correlated with systemic indices and modestly enriched for blood-culture—positive bacteremia (combined model AUC 0.707).

**Conclusions:**

Noninvasive salivary PTX3, calprotectin, and IL-8 are substantially elevated in early-onset neonatal pneumonia and mirror systemic inflammation. The three-marker panel showed near-excellent discrimination vs. healthy controls and modest enrichment for culture-positive bacteremia, suggesting value as an adjunct to bedside assessment in this case–healthy-control setting. Performance in neonates with non-infectious respiratory distress should be validated in prospective cohorts.

## Introduction

Neonatal infectious pneumonia is a major contributor to early morbidity and mortality. In 2019, pneumonia accounted for an estimated 740,000 deaths in children under five worldwide—14% of all under-five deaths and 22% among those aged 1–5 years (1). By time of presentation, neonatal infectious pneumonia is commonly classified as early- vs. late-onset, though definitions vary: ≤48 h after birth (2), ≤72 h (3), or up to 7 days (4). Early-onset neonatal pneumonia (EONP) is tightly linked to perinatal risk factors—prematurity, low birth weight, maternal chorioamnionitis, prolonged rupture of membranes, maternal *group B Streptococcus* (GBS) colonization, and intrapartum fever—and typically presents with respiratory distress at or soon after birth (1). Clinical manifestations often overlap with neonatal sepsis and may progress to shock or pulmonary complications (2). The pathogen spectrum mirrors that of early-onset sepsis, with GBS predominating in term infants and *Escherichia coli* in preterm infants; vertical transmission from maternal colonization (including GBS, *E. coli*, *Streptococcus pneumoniae*, and *Haemophilus influenzae*) is central (4–6).

Noninvasive, rapid biomarkers are needed to support early recognition and antibiotic decisions. Saliva—secretions from major/minor glands plus gingival crevicular fluid is easy and painless to obtain in neonates (7). PTX3 is a long pentraxin produced rapidly at infection sites by myeloid/barrier cells in response to cytokines and TLR signaling and has been detected in peripheral secretions including saliva (8–10). Calprotectin (S100A8/S100A9 heterodimer) is abundant in neutrophils/monocytes (11), and is measurable in multiple body fluids, including saliva (12–14). Interleukin-8 (CXCL8) drives neutrophil recruitment and activation (15) and shows salivary diagnostic utility across conditions (16–18). Evidence specific to EONP—particularly in non-intubated infants within early-onset windows- remains limited, motivating the current study.

We conducted a prospective case-control study to evaluate whether noninvasively collected salivary PTX3, calprotectin, and IL-8 can distinguish infants with clinically and radiographically confirmed EONP from healthy controls, correlate with conventional systemic inflammatory indices (hs-CRP, PCT, IL-6, leukocyte indices), and enrich for concomitant bacteremia among EONP cases.

## Methods and materials

### Ethical statement

This prospective case-control study complied with the Declaration of Helsinki and was approved by the ethics committee of the hospital. Written informed consent was obtained from the legal guardians of all participants. For healthy controls, only a single non-invasive salivary sample and routine clinical/delivery data were obtained; no additional procedures were performed.

### Sample size calculation

Because prior data for salivary biomarkers in EONP were unavailable, we used a precision-based approach informed by related neonatal pneumonia/sepsis studies reporting single-marker sensitivities/specificities around 0.75–0.90 (e.g., tracheal-aspirate presepsin in neonatal pneumonia (19); salivary CRP/platelet-based indices in late-onset neonatal pneumonia (20, 21). We targeted sensitivity and specificity of about 0.80 with 95% confidence-interval half-widths of 0.07–0.08 (two-sided *α* = 0.05). Standard formulas indicated a minimum of approximately 100 EONP cases and ∼120–130 controls to meet these precision goals; accordingly, we enrolled 100 EONP infants and 126 healthy controls. All enrolled participants provided analyzable saliva samples, so no additional allowance for dropout was required.

### Participants

A total of 226 neonates were enrolled between June 2022 and June 2025, including 100 neonates diagnosed with EONP (2, 22) and 126 healthy controls. All enrolled infants had a gestational age of at least 34 weeks and were delivered in the study hospital. EONP cases were included if they met all of the following criteria: (1) onset or progressive worsening of respiratory distress within 72 h after birth, including a sustained respiratory rate >60 breaths/min for ≥2 h and/or clinical signs such as grunting, nasal flaring, chest retractions, and an increased requirement for oxygen or respiratory support [e.g., supplemental oxygen, continuous positive airway pressure (CPAP), or mechanical ventilation] to maintain adequate oxygen saturation; (2) chest radiograph and/or lung ultrasound demonstrating new or progressive patchy or diffuse infiltrates, consolidation, or reticulonodular changes consistent with infectious pneumonia; (3) at least one laboratory indicator of infection: abnormal peripheral white blood cell count and/or neutrophil differential [e.g., neutropenia or neutrophilia, or an immature-to-total neutrophil ratio (I/T ratio) > 0.16], elevated C-reactive protein (CRP ≥ 10 mg/L), elevated procalcitonin (PCT ≥ 0.5 ng/mL), or a positive blood and/or airway culture yielding a pathogen compatible with the clinical presentation; (4) complete clinical and laboratory data available; and (5) salivary samples obtained before initiation of any systemic antibiotic therapy, with sampling performed within 2 h after the first definitive diagnosis. Infants with or without concomitant bacteremia and early-onset neonatal sepsis were eligible, provided that they fulfilled all of the above clinical and radiologic criteria for early-onset neonatal pneumonia.

Healthy controls were neonates who had no clinical signs of infection or respiratory distress within the first 72 h after birth and no abnormal findings on physical examination or routine in-hospital investigations. None of the healthy controls received systemic antibiotic therapy before or at the time of saliva sampling. Healthy controls had complete basic clinical data and provided adequate salivary samples within a postnatal time window comparable to that of the EONP group.

### Exclusion criteria

The following exclusion criteria were applied to both the EONP and healthy control groups: (1) a definite diagnosis of noninfectious pulmonary disease, such as classic respiratory distress syndrome (RDS) or transient tachypnea of the newborn (TTN), in which the clinical course and imaging findings were not compatible with infectious pneumonia; (2) presence of major congenital cardiopulmonary malformations or genetic syndromes (e.g., complex congenital heart disease, severe pulmonary hypoplasia, congenital diaphragmatic hernia) that could substantially affect respiratory function or confound outcome assessment; (3) severe perinatal asphyxia (e.g., 5 min Apgar score ≤3 and/or prolonged chest compressions with epinephrine during resuscitation) with persistent, marked multiorgan dysfunction, making it difficult to distinguish pneumonia-related respiratory failure from hypoxic*—*ischemic injury; (4) confirmed or strongly suspected purulent meningitis at admission, defined by markedly abnormal cerebrospinal fluid cell counts, protein and glucose levels, and/or a positive cerebrospinal fluid culture; (5) infants fulfilling the clinical diagnostic criteria for EONP but without radiologic evidence of pneumonia on chest radiograph and/or lung ultrasound; (6) failure to obtain a salivary sample or provision of a salivary sample of inadequate quality for analysis; (7) coexistence of other severe underlying diseases, such as severe primary immunodeficiency or active malignancy; and (8) refusal of parents or legal guardians to permit use of the infant's medical data or failure to obtain written informed consent.

### Saliva collection procedure

Salivary samples were collected after a definitive diagnosis of EONP had been established and before initiation of systemic antibiotic therapy. All sampling procedures were performed by neonatologists or neonatal nurses who had received standardized training. Before each procedure, hand hygiene was performed and sterile gloves were worn.

Oral samples were obtained using medical nylon flocked swabs (FLOQSwabs®, Copan, Brescia, Italy). In line with the approach described by Wunderlich et al. (23), and without interfering with clinical care, sampling was scheduled, whenever feasible, at least 30 min after the last feeding; in infants receiving donor human milk, sampling was preferably performed at least 60 min after feeding to minimize potential interference from residual breast milk or formula.

During collection, the infant's mouth was gently opened, and the swab was placed along the inner cheek and gumline on both sides. The swab was slowly rotated along the oral mucosa, approximately 5–10 rotations per side, for a total sampling time of about 10–20 s, avoiding the posterior tongue and pharynx to reduce gagging and discomfort. In infants with copious oral secretions, excess surface fluid was gently blotted with sterile gauze before swabbing.

Immediately after collection, the swab tip was placed into a pre-labeled 1.5 mL RNase-free microcentrifuge tube, the shaft was snapped off, and the tube was tightly capped. Then, 300–500 µL of prechilled normal saline was added to the tube and the contents were gently vortexed to ensure adequate elution of saliva into the preservation solution. All samples were temporarily stored at 4 °C and processed as soon as possible, within 4 h of collection, by centrifugation at 10,000 × g for 5 min at 4 °C. The supernatant was then aliquoted into RNase-free microcentrifuge tubes and stored at −80 °C until batch analysis. Each aliquot was thawed only once for biomarker measurement. Biomarker results are reported as concentrations in the eluted supernatant; because the original saliva volume absorbed by the swab was not directly quantified (e.g., by gravimetric weighing), the absolute concentrations should be interpreted as semi-quantitative.

### ELISA of salivary PTX3, calprotectin, and IL-8

Salivary PTX3, calprotectin, and IL-8 concentrations were measured using commercial ELISA kits according to the manufacturers' instructions: PTX3 with the Quantikine™ Human Pentraxin 3 Immunoassay (R&D Systems, Cat. DPTX30B); calprotectin with the Quantikine® Human Calprotectin Heterodimer Immunoassay (R&D Systems, Cat. DS8900); and IL-8 with the LEGEND MAX™ High Sensitivity Human IL-8 ELISA Kit (BioLegend, Cat. 431517).

For PTX3, the limit of detection was approximately 0.014 ng/mL, with a calibration range of 0.313–20 ng/mL, and intra- and inter-assay coefficients of variation of 1.2%–2.6% and 4.4%–6.9%, respectively. For calprotectin, the limit of detection was approximately 0.086 ng/mL, the calibration range was 0.625–40 ng/mL, and intra- and inter-assay coefficients of variation were 2.7%–4.5% and 3.2%–5.8%, respectively. For IL-8, the limit of detection was approximately 0.235 pg/mL, the calibration range was 0.78–50 pg/mL, and intra- and inter-assay coefficients of variation were 4.3%–5.3% and 5.7%–6.3%, respectively.

All standards and samples were assayed in duplicate. Laboratory personnel performing the ELISA assays were blinded to the clinical status and group allocation of the infants.

### Statistical analysis

Continuous variables were assessed for distribution (Shapiro–Wilk). Skewed data are presented as median (IQR) and compared with the Mann–Whitney *U* test; categorical variables are presented as *n* (%) and compared with the *χ*^2^ test or Fisher's exact test, as appropriate. Associations were examined using Spearman rank correlations. To assess whether salivary biomarkers were independently associated with EONP after accounting for potential confounders, we fitted a multivariable binary logistic regression model with EONP status as the dependent variable. Predictors included salivary biomarkers, with adjustment for gestational age and maternal intrapartum intravenous antibiotic prophylaxis. Diagnostic performance was evaluated using receiver operating characteristic (ROC) curve analysis, with discrimination quantified by the area under the ROC curve (AUC) and its 95% confidence interval (CI). For single biomarkers, the optimal cutoff value was selected by maximizing the Youden index (J = sensitivity + specificity−1). At the chosen cutoff, we report sensitivity and specificity, positive and negative likelihood ratios (LR+ and LR−), and positive and negative predictive values (PPV and NPV). AUCs and 95% CIs were estimated using the DeLong method. A combined model incorporating salivary PTX3, calprotectin, and IL-8 was developed using multivariable logistic regression to generate individual predicted probabilities. Apparent (development) performance was calculated by fitting the logistic regression model on the full development dataset and computing the ROC/AUC based on the resulting in-sample predicted probabilities. Internal validation was performed using out-of-fold predicted probabilities obtained from leave-one-out cross-validation (LOOCV) and 5-fold cross-validation. All tests were two-sided, with a significance level of *α* = 0.05. Analyses were performed using SPSS Statistics version 22.0 (IBM Corp.) and GraphPad Prism version 8.

## Results

### Baseline characteristics of neonates with EONP and healthy controls

The EONP and healthy control groups were broadly comparable in maternal and neonatal baseline characteristics (Table 1), including maternal age, parity, gestational diabetes, hypertensive disorders of pregnancy, gestational age, birth weight, sex, mode of delivery, 5 min Apgar scores, postnatal age at saliva sampling, and axillary temperature (all *P* *>* 0.05). As expected, intrapartum risk factors for early-onset infection were more frequent in the EONP group. Clinical chorioamnionitis was present in 25.0% of EONP pregnancies vs. 3.2% of healthy controls, intrapartum fever ≥38 °C in 22.0% vs. 2.4%, and premature rupture of membranes ≥18 h in 28.0% vs. 5.6% (all *P* *<* 0.001), respectively. Intrapartum intravenous antibiotic prophylaxis was administered to 52.0% of mothers in the EONP group and 23.8% in the healthy control group (*P* *<* 0.001). At the time of saliva sampling, infants with EONP had higher respiratory rates (72 breaths/min *vs.* 47 breaths/min) and heart rates (140 beats/min *vs.* 130 beats/min), and lower room-air SpO_2_ (89% *vs.* 98%) than healthy controls (all *P* *<* 0.001), consistent with their underlying respiratory compromise.

**Table 1:** Baseline maternal and neonatal characteristics of EONP cases and healthy controls.

Variable	EONP cases (*n* = 100)	Healthy controls (*n* = 126)	*p*-value
Perinatal and maternal characteristics			
Maternal age, years	30 (26*–*35)	31 (27.75*–*37)	0.265
Primiparity	55 (55.0)	61 (48.4)	0.350
Gestational diabetes	10 (10.0)	16 (12.7)	0.833
Hypertensive disorders of pregnancy	12 (12.0)	21 (16.7)	0.351
Clinical chorioamnionitis	25 (25.0)	4 (3.2)	< 0.001
Intrapartum fever ≥38 °C	22 (22.0)	3 (2.4)	<0.001
Premature rupture of membranes ≥18 h	28 (28.0)	7 (5.6)	<0.001
Intrapartum IV antibiotic prophylaxis	52 (52.0)	30 (23.8)	<0.001
Mode of delivery			0.891
Vaginal delivery	62 (62.0)	76 (60.3)	
Cesarean section	38 (38.0)	50 (39.7)	
Neonatal characteristics			
Gestational age category			0.091
34*–*36^+6^ weeks	20 (20.0)	14 (11.1)	
≥37 weeks	80 (80.0)	112 (88.9)	
Birth weight, g	2,733 (2,612*–*2,838)	2,753 (2,635*–*2,899)	0.148
Male sex	60 (60.0)	87 (69.0)	0.163
5 min Apgar score	8 (7*–*9.75)	9 (8*–*10)	0.358
Clinical status at saliva sampling			
Postnatal age at sampling, h	41.75 (29.33*–*51.00)	37.90 (26.08*–*47.65)	0.182
Axillary temperature, °C	37.1 (36.4*–*37.6)	36.8 (36.5*–*37.3)	0.067
Respiratory rate, breaths/min	72 (66*–*79)	47 (42*–*51)	<0.001
Heart rate, beats/min	140 (123*–*157)	130 (115*–*141)	<0.001
Room-air SpO_2_, %	89 (87*–*93)	98 (97*–*100)	<0.001

Data are presented as median (IQR), mean ± SD, or *n* (%), as appropriate.

### Clinical signs, laboratory, microbiological, and imaging findings in infants with EONP

In the EONP group (Table 2), signs of respiratory distress were frequent: nasal flaring in 75/100 infants (75.0%), chest wall retractions in 73 (73.0%), grunting in 62 (62.0%), and central cyanosis in 21 (21.0%). Inflammatory and hematologic markers were clearly elevated. Median high-sensitivity CRP was 45.0 mg/L (IQR 32.45*–*58.50), serum procalcitonin 4.65 ng/mL (2.90*–*7.10), white blood cell counts 25.5 × 10^9^/L (17.45*–*31.65), and absolute neutrophil count 12.85 × 10^9^/L (9.50*–*17.55). The immature-to-total neutrophil ratio had a median of 0.27 (0.20*–*0.36). Median platelet count was 182.0 × 10^9^/L (122.5*–*240.0), and median serum IL-6 concentration was 239.2 pg/mL (160.4*–*318.7).

**Table 2:** Clinical signs, laboratory indices, microbiology, and imaging findings in infants with EONP.

Variable	Value
Signs of respiratory distress	
Grunting	62 (62.0)
Nasal flaring	75 (75.0)
Chest wall retractions	73 (73.0)
Central cyanosis	21 (21.0)
Inflammatory and hematologic markers	
High-sensitivity C-reactive protein (CRP), mg/L	45.0 (32.45*–*58.50)
Serum Procalcitonin (PCT), ng/mL	4.65 (2.90*–*7.10)
White blood cell count (WBC), ×10^9^/L	25.5 (17.45*–*31.65)
Absolute neutrophil count (ANC), ×10^9^/L	12.85 (9.50*–*17.55)
Immature-to-total neutrophil (I/T) ratio	0.27 (0.20*–*0.36)
Platelet count, ×10^9^/L	182.0 (122.5*–*240.0)
Serum interleukin-6 (IL-6), pg/mL	239.2 (160.4*–*318.7)
Microbiology	
Positive blood culture	34 (34.0)
Positive upper airway bacterial culture	37 (37.0)
At least one pathogen detected by PCR	44 (44.0)
Chest radiograph pattern	
Patchy infiltrates	60 (60.0)
Diffuse infiltrates	22 (22.0)
Consolidation	32 (32.0)
Reticulonodular changes	18 (18.0)

Data are presented as median (IQR), mean ± SD, or *n* (%), as appropriate.

Microbiologically, 34 infants (34.0%) had a positive blood culture, 37 (37.0%) had a positive upper airway bacterial culture, and respiratory pathogen PCR identified at least one pathogen in 44 (44.0%) cases. Chest radiographs most often showed patchy infiltrates in 60 infants (60.0%), with diffuse infiltrates in 22 (22.0%), consolidation in 32 (32.0%), and reticulonodular changes in 18 (18.0%); some infants had more than one radiographic pattern.

### Salivary PTX3, calprotectin, and IL-8 are markedly elevated in EONP

**Figure 1:**
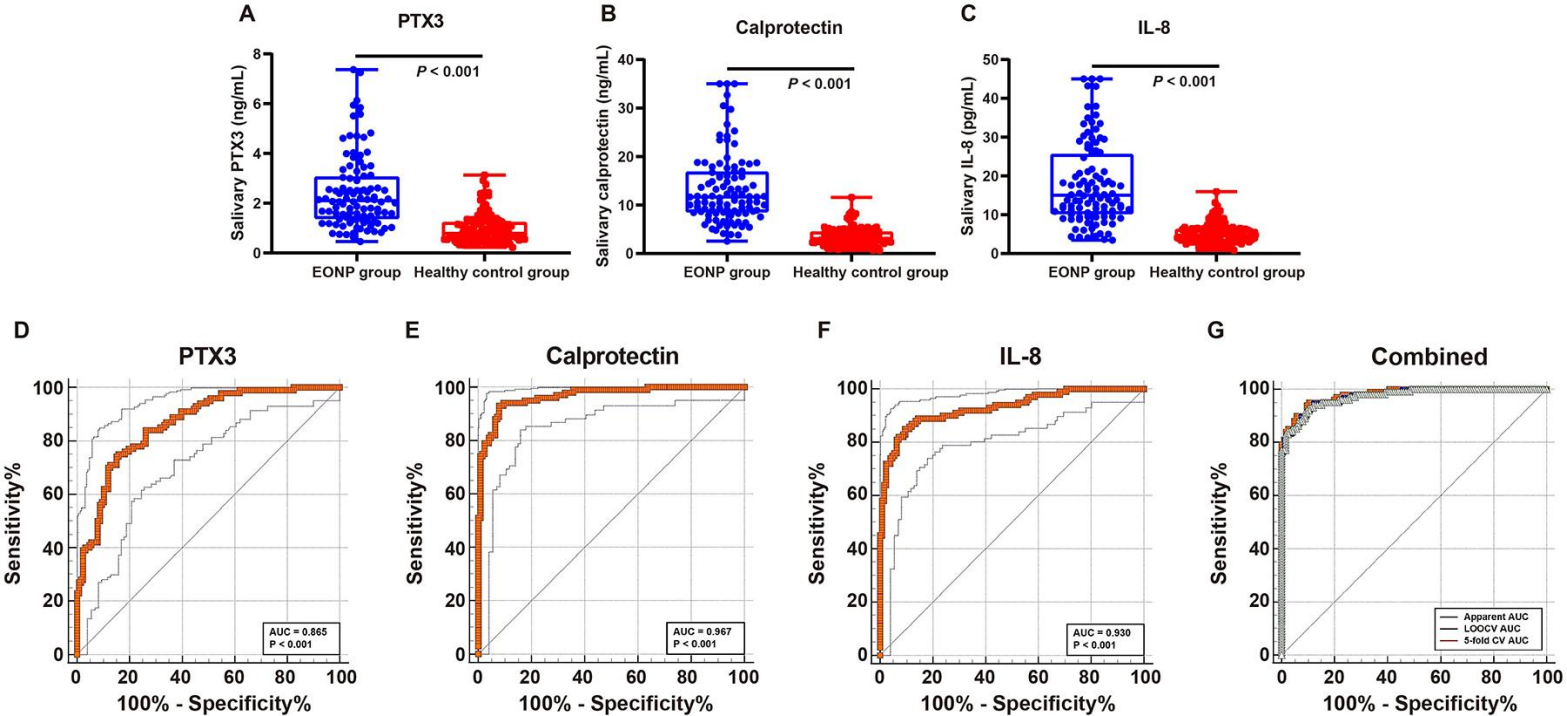
Salivary PTX3, calprotectin, and IL-8 are elevated in early-onset neonatal pneumonia (EONP) and show strong case—control discrimination. **(A*–*C)** Distributions of salivary PTX3, calprotectin, and IL-8 in EONP *vs.* healthy controls (medians with IQRs; Mann–Whitney tests, all *P* < 0.001). **(D*–*F)** ROC curves for each single biomarker with optimal cutoffs (PTX3 ≥ 1.38 ng/mL; calprotectin ≥5.49 ng/mL; IL-8 ≥ 8.69 pg/mL). The solid line denotes the ROC curve and the dashed lines indicate the 95% confidence interval (lower and upper limits). **(G)** ROC curves for the three-marker combined model (logistic regression using PTX3, calprotectin, and IL-8). The combined model achieved an apparent AUC of 0.978, and internally validated performance is overlaid (LOOCV and 5-fold cross-validation), demonstrating minimal performance degradation.

Compared with healthy controls, infants with EONP had substantially higher salivary PTX3, calprotectin, and IL-8 levels (Figures 1A–C). For PTX3, the median concentration in the EONP group was 2.11 ng/mL (IQR 1.36*–*3.08) vs. 0.79 ng/mL (IQR 0.52*–*1.25) in healthy controls (Mann–Whitney *P* *<* 0.001). For calprotectin, the median was 11.65 ng/mL (IQR 8.47*–*16.93) in EONP infants and 3.07 ng/mL (IQR 2.09*–*4.53) in healthy controls (*P* *<* 0.001). For IL-8, the median was 15.02 pg/mL (IQR 10.16*–*25.71) in the EONP group compared with 4.67 pg/mL (IQR 3.11*–*6.34) in healthy controls (*P* *<* 0.001). Together, these findings indicate that all three salivary biomarkers are significantly elevated in EONP and show substantial separation between cases and healthy controls. A multivariable binary logistic regression model was fitted to evaluate whether the salivary biomarkers were independently associated with EONP after adjustment for gestational age and maternal intrapartum IV antibiotic prophylaxis (Supplementary Table S1). After adjustment, salivary calprotectin remained an independent predictor of EONP (B = 0.772; adjusted OR = 2.163, 95% CI 1.639–2.855; *P* *<* 0.001), as did salivary IL-8 (B = 0.404; adjusted OR = 1.497, 95% CI 1.226–1.828; *P* *<* 0.001). In contrast, salivary PTX3 was not statistically significant in the adjusted model (B = 0.446; adjusted OR = 1.562, 95% CI 0.537–4.544; *P* *=* 0.413). Neither gestational age category (adjusted OR = 1.410, 95% CI 0.196–10.159; *P* *=* 0.733) nor intrapartum IV antibiotic prophylaxis (adjusted OR = 2.615, 95% CI 0.543–12.599; *P* *=* 0.231) was significant in this model. To assess robustness, we additionally performed bootstrap resampling (1,000 samples). The bootstrap results were consistent with the primary analysis: the regression coefficients for calprotectin (bootstrap B 95% CI 0.573–1.262; *P* *=* 0.001) and IL-8 (bootstrap B 95% CI 0.232–0.710; *P* *=* 0.001) remained significant and their confidence intervals did not cross zero, supporting the stability of these associations. In contrast, PTX3 did not show a significant association on bootstrap validation (bootstrap *P* *=* 0.350).

### Diagnostic performance of salivary biomarkers for discriminating EONP from healthy controls

Using ROC-derived optimal cutoffs, all three salivary biomarkers showed good to excellent diagnostic performance for identifying EONP from healthy controls (Figures 1D–F, Table 3). For PTX3 (cutoff ≥ 1.38 ng/mL), the AUC was 0.865, with a sensitivity of 75% and a specificity of 84.1% (Youden index 0.59). The corresponding positive and negative likelihood ratios were 4.7 and 0.30, and the positive and negative predictive values in this cohort were approximately 78.9% and 80.9%, respectively. Salivary calprotectin (cutoff ≥ 5.49 ng/mL) yielded the highest diagnostic accuracy among single markers, with an AUC of 0.967, sensitivity 93%, specificity 92.1%, and a Youden index of 0.85. The positive and negative likelihood ratios were 11.8 and 0.08, with a PPV of about 90.3% and an NPV of 94.3%. For IL-8 (cutoff ≥ 8.69 pg/mL), the AUC was 0.930, with a sensitivity of 85% and specificity of 90.5% (Youden index 0.76). The positive and negative likelihood ratios were 9.0 and 0.17, and the corresponding PPV and NPV were 87.7% and 88.4%. A multivariable logistic regression model combining salivary PTX3, calprotectin, and IL-8 showed excellent discrimination for identifying EONP in the overall cohort (Figure 1G). The apparent AUC estimated on the development dataset was 0.978. Bootstrap resampling (1,000 iterations) yielded a mean AUC of 0.979 with a percentile 95% confidence interval of 0.963–0.991. The mean optimism estimated by bootstrap was 0.0015, resulting in an optimism-corrected AUC of 0.976, indicating minimal optimism. Consistent results were obtained using cross-validation: the LOOCV AUC was 0.974, and the 5-fold cross-validated AUC was 0.972 ± 0.024. Collectively, these findings support stable model discrimination with limited evidence of overfitting.

**Table 3:** Diagnostic performance of salivary PTX3, calprotectin, and IL-8 for early-onset neonatal pneumonia and for culture-positive bacteremia within EONP.

ROC	EONP cases *vs.* Healthy controls			Blood-culture positive ***vs.*** negative bacteremia within EONP		
	PTX3	Calprotectin	IL-8	PTX3	Calprotectin	IL-8
Cutoff value	≥1.38 ng/mL	≥5.49 ng/mL	≥8.69 pg/mL	≥2.51 ng/mL	≥14.57 ng/mL	≥18.97 pg/mL
AUC	0.865	0.967	0.93	0.725	0.682	0.689
Sensitivity (%)	75	93	85	64.71	55.88	58.82
Specificity (%)	84.1	92.1	90.5	80.3	78.79	80.3
Youden index	0.59	0.85	0.76	0.45	0.347	0.391
LR+	4.72	11.77	8.95	3.28	2.63	2.99
LR–	0.3	0.08	0.17	0.44	0.56	0.51
PPV (%)	78.9	90.3	87.7	62.9	57.6	60.6
NPV (%)	80.9	94.3	88.4	81.5	77.6	79.1

Cutoffs selected by Youden index on ROC analyses; AUC, area under ROC curve; LR+, positive likelihood ratio; LR−, negative likelihood ratio; PPV, %, positive predictive value; NPV, %, negative predictive value.

### Salivary biomarkers are tightly intercorrelated and track systemic inflammation in EONP

**Figure 2:**
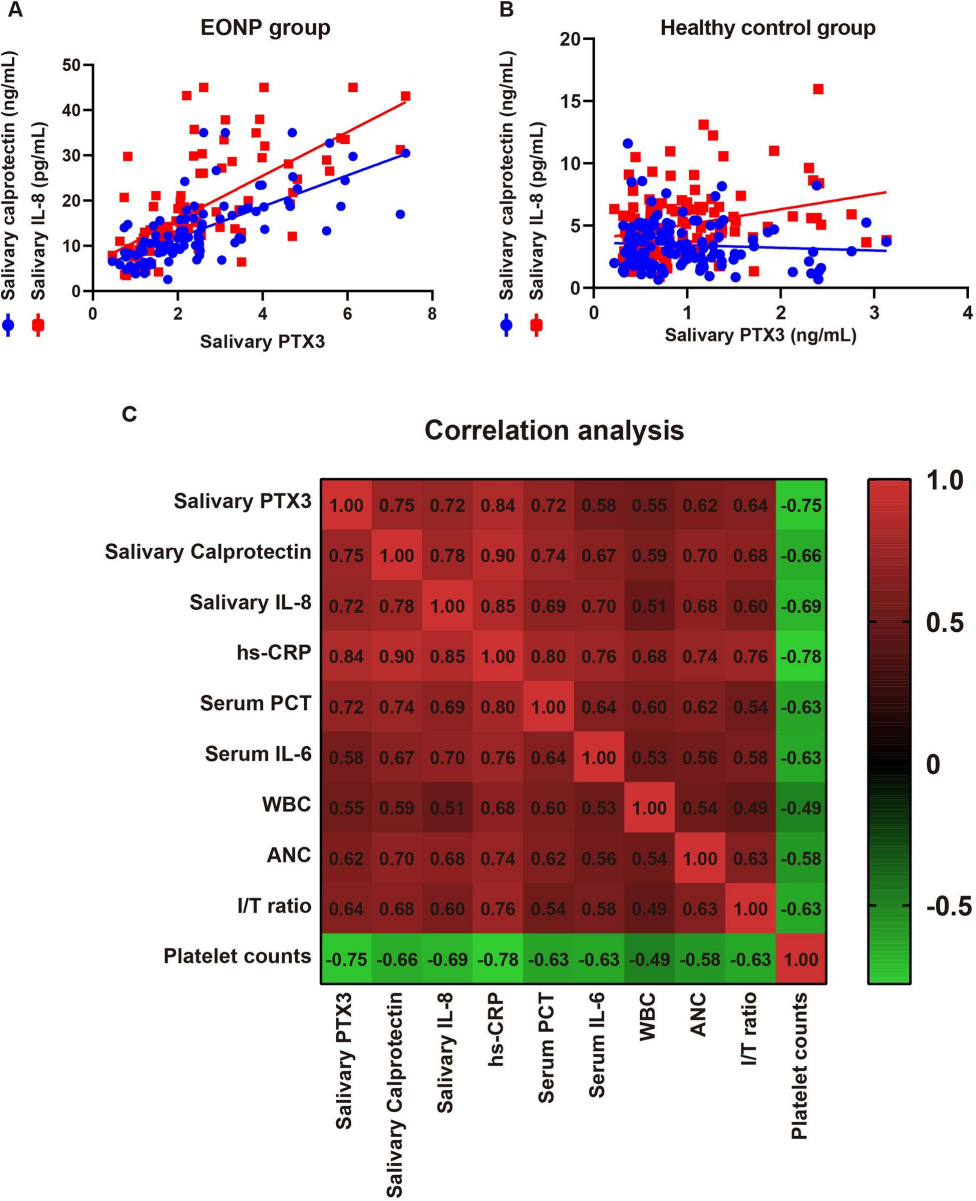
Salivary biomarkers are inter-correlated and track systemic inflammation in EONP. **(A)** Pairwise correlations among salivary PTX3, calprotectin, and IL-8 in EONP (Spearman *r*, all *P* *<* 0.001). **(B)** Corresponding correlations in healthy controls (all *P* > 0.05). **(C)** Heatmap of Spearman correlations between salivary biomarkers and systemic indices (hs-CRP, serum PCT, serum IL-6, WBC, ANC, I/T ratio, platelets) in EONP, showing moderate-to-strong positive associations with inflammatory markers and inverse associations with platelets (panel labels show exact *r*).

Salivary biomarkers were strongly inter-correlated (PTX3—calprotectin *r* = 0.75; PTX3—IL-8 *r* = 0.72; calprotectin—IL-8 *r* = 0.78, all *P* *<* 0.001; Figure 2A), whereas no significant correlations were observed among these markers in the healthy control group (all *P* > 0.05; Figure 2B). Each marker tracked systemic inflammation (all *P* *<* 0.05; Figure 2C), showing the highest correlations with hs-CRP (PTX3 *r* = 0.84; calprotectin *r* = 0.90; IL-8 *r* = 0.85), and moderate—strong correlations with PCT (0.72, 0.74, 0.69) and IL-6 (0.58, 0.67, 0.70). Associations with leukocyte indices were moderate (WBC 0.55, 0.59, 0.51; ANC 0.62, 0.70, 0.68; I/T ratio 0.64, 0.68, 0.60). All three were inversely related to platelet count with relatively strong negative correlations (−0.75, −0.66, −0.69), supporting that salivary PTX3, calprotectin, and IL-8 mirror systemic inflammatory burden.

### Salivary markers modestly enrich for bacteremia among EONP

**Figure 3:**
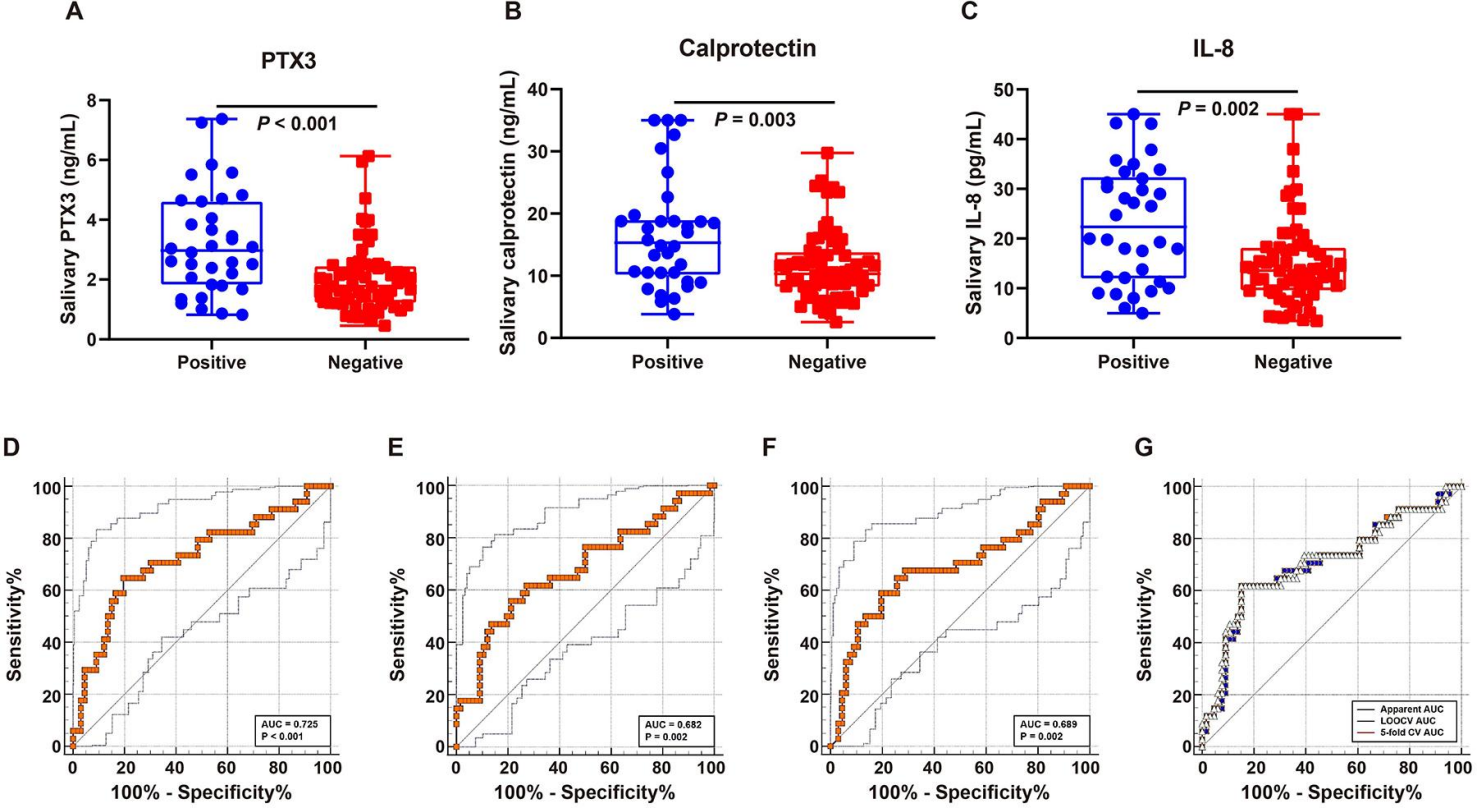
Salivary biomarkers modestly enrich for blood-culture-positive bacteremia within EONP. **(A*–*C)** Salivary PTX3, calprotectin, and IL-8 in culture-positive vs. culture-negative EONP cases (medians with IQRs; Mann–Whitney *P* *<* 0.001, =0.003, =0.002, respectively). **(D*–*F)** ROC curves for bacteremia detection using single salivary markers with optimal cutoffs (PTX3 ≥ 2.51 ng/mL; calprotectin ≥14.57 ng/mL; IL-8 ≥ 18.97 pg/mL), yielding AUCs 0.725, 0.682, and 0.689, respectively. The solid line denotes the ROC curve and the dashed lines indicate the 95% confidence interval (lower and upper limits). **(G)** ROC curves for the three-marker combined model (logistic regression using PTX3, calprotectin, and IL-8). The combined model achieved an apparent AUC of 0.702, with internally validated AUCs of 0.706 (5-fold cross-validation) and 0.702 (LOOCV), indicating stable but moderate enrichment performance.

Among infants with EONP, salivary PTX3, calprotectin, and IL-8 were higher in culture-positive than culture-negative cases (Figures 3A–C). Median (IQR) PTX3 was 2.97 (1.82–4.62) ng/mL *vs.* 1.78 (1.22–2.46) ng/mL (Mann–Whitney *P* *<* 0.001); calprotectin 15.30 (10.12–19.02) ng/mL *vs.* 10.31 (8.17–13.84) ng/mL (*P* *=* 0.003); and IL-8 22.34 (11.90–32.42) pg/mL *vs.* 13.58 (9.50–18.24) pg/mL (*P* *=* 0.002). In EONP infants, salivary biomarkers showed fair discrimination for blood-culture—positive bacteremia. Using ROC-optimized thresholds (PTX3 ≥ 2.51 ng/mL; calprotectin ≥14.57 ng/mL; IL-8 ≥ 18.97 pg/mL, Figures 3D–F, Table 3), performance estimates were: PTX3 AUC = 0.725, sensitivity = 64.71%, specificity = 80.30%; calprotectin AUC = 0.682, sensitivity = 55.88%, specificity = 78.79%; IL-8 AUC = 0.689, sensitivity = 58.82%, specificity = 80.30%). Using the combined salivary biomarker model score to discriminate culture-positive from culture-negative EONP cases demonstrated moderate performance (Figure 3G). The AUC was 0.702 using the apparent predicted probability. Internally validated predictions showed nearly identical discrimination, with an AUC of 0.706 for 5-fold cross-validated probabilities and 0.702 for LOOCV probabilities, supporting the robustness of this enrichment signal. These findings suggest that, within confirmed EONP, the salivary panel may help enrich for culture-positive cases, although discrimination is limited compared with its performance for EONP detection.

## Discussion

This study shows three key findings. First, noninvasive salivary PTX3, calprotectin, and IL-8 were substantially higher in EONP than in healthy controls and each showed good-to-excellent case-control discrimination, while a three-marker model achieved near-excellent AUC (0.978). Second, within EONP, salivary biomarkers tracked systemic inflammation (moderate—strong correlations with hs-CRP, IL-6, PCT, leukocyte indices, and inverse associations with platelets). Third, salivary markers modestly enriched for blood-culture—positive bacteremia, with the combined model outperforming single markers for this purpose (EONP-only AUC = 0.702 across apparent and internally validated predictions).

Many neonatal sepsis frameworks operationalize early-onset disease as presentation within ≤72 h, aligning with vertical exposure biology (chorioamnionitis, prolonged membrane rupture, intrapartum fever, maternal GBS colonization) and with contemporary clinical pathways (24, 25). Including infants with concomitant bacteremia reflects real-world EONP, where pulmonary infection and systemic invasion exist on a spectrum governed by the same perinatal exposures; stratified analyses by culture status preserve generalizability while showing that salivary signals rise with pathogen-proven disease. Accordingly, the culture-stratified results support a severity-gradient interpretation, in which higher salivary inflammatory signals accompany pathogen-proven systemic invasion rather than merely non-specific stress.

The biological plausibility for elevated salivary PTX3, calprotectin, and IL-8 in EONP is strong. PTX3 is an acute-phase pattern-recognition molecule produced by leukocytes, endothelial and stromal cells after TLR/cytokine engagement and rises within hours in infectious inflammation; it is detectable in saliva, albeit with context-dependent concordance to systemic markers (8–10). In community adults, salivary PTX3 shows limited correlation with systemic inflammation (9). In acute EONP, a higher inflammatory burden and barrier-site involvement likely synchronize mucosal and systemic responses, yielding moderate—strong saliva—blood correlations—a pathophysiologic difference rather than an assay artifact. Calprotectin (S100A8/A9) is the dominant neutrophil alarmin and a proxy for mucosal neutrophil load, abundant in saliva given neutrophils as a major salivary cell population (26). IL-8 (CXCL8) orchestrates neutrophil chemotaxis and degranulation, amplifying barrier-site inflammation (27). IL-8 levels could increase 10- to 100-fold above normal range in response to other inflammatory cytokines, such as IL-1 and tumour necrosis factor-α (TNF-α), as well as cellular stress and viral and bacterial process (28). In EONP, airway/mucosal neutrophilic inflammation plus increased vascular permeability can spill these mediators into oral fluids, explaining clear case—control separation, correlations with systemic indices, and higher levels in culture-positive cases. Taken together, complement engagement by PTX3, neutrophil activation reflected by calprotectin, and IL-8-driven recruitment provides mechanistic coherence for the observed associations with hs-CRP, IL-6, PCT, leukocytosis and I/T ratio, and with platelet consumption/redistribution in inflammation (27, 29, 30).

Our single-marker AUCs (0.865–0.967) are comparable to, or exceed, many blood-based inflammatory indices reported for early-onset disease; the combined salivary model (AUC 0.978) approaches best-in-class performance reported in pediatric infectious settings, while offering noninvasive, bedside feasibility. Prior neonatal work emphasizing blood or tracheal aspirate markers (19, 31) supports the construct that innate-immune mediators discriminate infection; our data extend this to a painless saliva platform suitable for neonates. To reduce overfitting concerns, we performed internal validation; discrimination remained stable across bootstrap resampling, LOOCV, and 5-fold cross-validation, indicating minimal optimism and supporting model robustness.

Beyond EONP case identification, the bacteremia enrichment analysis provides clinically relevant insight. Blood-culture positivity likely reflects higher bacterial burden and/or greater systemic invasion, which should intensify innate immune activation and neutrophil trafficking. Therefore, a salivary panel dominated by PTX3, calprotectin, and IL-8 would be expected to increase in pathogen-proven cases. Consistent with this biology, within confirmed EONP, the combined model achieved moderate discrimination for blood-culture positivity (AUC ≈ 0.70), suggesting these salivary markers may track bacterial burden/systemic invasion-related inflammation rather than general inflammation alone. Although insufficient to replace blood culture, this signal may be useful for risk stratification or “enrichment” of infants more likely to have bacteremia who may warrant closer monitoring and expedited evaluation.

Several limitations should be considered when interpreting the diagnostic performance of the salivary biomarkers. First, First, the control group comprised healthy neonates, and we did not include symptomatic “sick controls” with non-infectious respiratory distress [e.g., respiratory distress syndrome [RDS], transient tachypnea of the newborn (TTN), meconium aspiration]. This case–control spectrum may introduce spectrum bias and potentially overestimate discrimination metrics (e.g., AUC); therefore, the biomarkers may partly capture non-specific physiological stress or inflammation rather than pneumonia-specific processes. Accordingly, we cannot exclude stress- or hypoxemia-associated elevations of PTX3, calprotectin, and IL-8 in neonates with non-infectious respiratory distress, which may reduce specificity in the real-world dyspnea differential-diagnosis setting. Second, this was a single-center case–control study, and saliva was collected after EONP had been clinically confirmed and prior to initiation of systemic antibiotic therapy; thus, diagnostic performance at the initial point of clinical suspicion (before the diagnostic work-up and treatment decisions) remains unknown. Third, our cohort predominantly comprised neonates of ≥34 weeks' gestation, limiting generalizability to very preterm or critically ill neonates. Fourth, although maternal intrapartum antibiotic prophylaxis and gestational age could influence inflammatory readouts, multivariable analyses adjusting for these covariates yielded materially similar estimates for the key biomarkers, supporting internal robustness; nevertheless, residual confounding cannot be excluded. Fifth, the optimal cut-off values were derived using the Youden index in the current single-center dataset and should be considered exploratory. These thresholds require external validation in independent cohorts (ideally including symptomatic non-infectious respiratory distress controls) before clinical implementation. Sixth, saliva was collected using oral swabs and eluted into 300–500 μL of prechilled normal saline. Because the volume of saliva absorbed by the swab likely varies across neonates, we did not directly quantify the original saliva volume (e.g., by pre-/post-collection gravimetric weighing) to correct for individual dilution factors. Accordingly, the reported concentrations represent eluate concentrations and should be interpreted as semi-quantitative, with potential contribution from variability in sample volume and elution efficiency. To mitigate this, we applied a standardized sampling protocol (consistent swab type, collection duration/site, and processing workflow) across all participants. Future studies should incorporate objective volume normalization (gravimetric weighing, total protein normalization, or collection devices enabling volumetric sampling) and validate thresholds across independent cohorts prior to clinical implementation. Taken together, given post-diagnosis sampling and a single-center case–control design, these findings should be interpreted primarily as biomarker expression differences with exploratory discrimination, rather than definitive first-presentation diagnostic performance. Future multicenter prospective studies should collect samples at first clinical suspicion and include non-infectious respiratory distress comparators (e.g., RDS/TTN/meconium aspiration) to validate clinical utility and establish generalizable thresholds.

## Conclusion

Noninvasive salivary PTX3, calprotectin, and IL-8 were substantially higher in early-onset neonatal pneumonia than in healthy controls, showed good-to-excellent case-control discrimination, correlated with systemic inflammatory indices, and modestly enriched for culture-positive bacteremia—with a three-marker model performing best. These findings support feasibility and exploratory discrimination against healthy controls; validation against non-infectious respiratory distress comparators at first clinical suspicion is warranted.

## Data Availability

The raw data supporting the conclusions of this article will be made available by the authors, without undue reservation.
