# Supplementary material for: Noninvasive salivary biomarkers (PTX3, calprotectin, and IL-8) for early-onset neonatal pneumonia: case-control differences and exploratory discrimination
Source: Front Pediatr. 2026 Mar 4;14:1747967. doi: 10.3389/fped.2026.1747967 (PMC12996215; doi:10.3389/fped.2026.1747967)
Supplement: Supplementary file 1 [file Table1.docx]

**Supplementary Table 1. Multivariable logistic regression and bootstrap validation for predictors of EONP**

| **Predictor** | **Salivary PTX3** | **Salivary Calprotectin** | **Salivary IL-8** | **Gestational age (≥ 37 vs 34–36⁺⁶)** | **Intrapartum IV antibiotic prophylaxis (yes vs no)** |
| --- | --- | --- | --- | --- | --- |
| B | 0.446 | 0.772 | 0.404 | 0.344 | 0.961 |
| S.E. | 0.545 | 0.142 | 0.102 | 1.008 | 0.802 |
| Wald | 0.67 | 29.686 | 15.73 | 0.116 | 1.435 |
| *P* | 0.413 | <0.001 | <0.001 | 0.733 | 0.231 |
| Adjusted OR | 1.562 | 2.163 | 1.497 | 1.41 | 2.615 |
| 95% CI for OR | 0.537–4.544 | 1.639–2.855 | 1.226–1.828 | 0.196–10.159 | 0.543–12.599 |
| **Bootstrap validation** |  |  |  |  |  |
| B | 0.446 | 0.772 | 0.404 | 0.344 | 0.961 |
| S.E. | 0.537 | 0.176 | 0.113 | 0.939 | 0.867 |
| *P* | 0.35 | 0.001 | 0.001 | 0.669 | 0.172 |
| 95% CI for B | -0.632–1.560 | 0.573–1.262 | 0.232–0.710 | -1.652–1.974 | -0.344–3.187 |
| 95% CI for OR * | 0.53–4.76 | 1.77–3.53 | 1.26–2.03 | 0.190–7.200 | 0.710–24.220 |

Notes: Bootstrap: 1000 samples. *Bootstrap OR 95% CI calculated as exp (lower B) to exp (upper B).
